# Dysregulation of S-adenosylmethionine Metabolism in Nonalcoholic Steatohepatitis Leads to Polyamine Flux and Oxidative Stress

**DOI:** 10.3390/ijms23041986

**Published:** 2022-02-11

**Authors:** Connor Quinn, Mario C. Rico, Carmen Merali, Salim Merali

**Affiliations:** Department of Pharmaceutical Sciences, School of Pharmacy, Temple University, Philadelphia, PA 19122, USA; connor.quinn1@temple.edu (C.Q.); mario.rico@temple.edu (M.C.R.); carmen.merali@temple.edu (C.M.)

**Keywords:** nonalcoholic steatohepatitis, polyamines, glycine-*N*-methyl transferase, S-adenosylmethionine

## Abstract

Nonalcoholic fatty liver disease (NAFLD) is the number one cause of chronic liver disease worldwide, with 25% of these patients developing nonalcoholic steatohepatitis (NASH). NASH significantly increases the risk of cirrhosis and decompensated liver failure. Past studies in rodent models have shown that glycine-*N*-methyltransferase (GNMT) knockout results in rapid steatosis, fibrosis, and hepatocellular carcinoma progression. However, the attenuation of GNMT in subjects with NASH and the molecular basis for its impact on the disease process is still unclear. To address this knowledge gap, we show the reduction of GNMT protein levels in the liver of NASH subjects compared to healthy controls. To gain insight into the impact of decreased GNMT in the disease process, we performed global label-free proteome studies on the livers from a murine modified amylin diet-based model of NASH. Histological and molecular characterization of the animal model demonstrate a high resemblance to human disease. We found that a reduction of GNMT leads to a significant increase in S-adenosylmethionine (AdoMet), an essential metabolite for transmethylation reactions and a substrate for polyamine synthesis. Further targeted proteomic and metabolomic studies demonstrated a decrease in GNMT transmethylation, increased flux through the polyamine pathway, and increased oxidative stress production contributing to NASH pathogenesis.

## 1. Introduction

Nonalcoholic fatty liver disease (NAFLD) is an excess buildup of fat in the liver and is the leading cause of chronic liver disease worldwide. Currently, it is estimated that 25% of the human population has NAFLD, and of these patients, 25% will progress to the more severe debilitating disease, nonalcoholic steatohepatitis (NASH) [1,2]. NASH is characterized by hepatocellular damage involving steatosis, inflammation, and liver fibrosis. It is a significant risk factor for cirrhosis and hepatocellular carcinoma, and it significantly increases the risk of death due to liver-related causes [1,2]. Currently, there are no FDA-approved treatments on the market for treating NASH and current therapies are limited to lifestyle interventions (calorie restriction, exercise) and treating comorbidities (insulin resistance, obesity). Due to low patient compliance and the growing obesity epidemic, the number of patients diagnosed with NAFLD and NASH continues to grow [3].

The pathogenesis of NASH has been described by the “multiple hit theory”, where several metabolic insults, including increased de novo lipogenesis, mitochondrial dysfunction, inflammation, and oxidative stress, simultaneously lead to the disease state [4]. The molecular mechanisms driving these insults are still not well understood. Glycine-*N*-methyl transferase (GNMT) is the most abundant methyltransferase in the liver, accounting for over 1% of all cytosolic protein in hepatocytes [5,6]. It catalyzes the transfer of a methyl group from S-adenosylmethionine (AdoMet), the universal methyl donor, to glycine to form sarcosine. Because of its high abundance, GNMT plays a crucial role in controlling AdoMet metabolism and methyltransferase reactions involving AdoMet. Furthermore, 75% of all transmethylation reactions occur in the liver, making AdoMet a vital component of liver metabolism and regulation [5]. The importance of GNMT in the progression of NAFLD has been demonstrated in several studies. GNMT KO animals spontaneously develop steatosis around three months of age and inflammation and fibrosis of the liver at eight months [7,8]. These studies indicated GNMT KO results in a significant increase in liver AdoMet that is directly responsible for steatosis and fibrosis. It was shown that elevated AdoMet could lead to hypermethylation of DNA and modulation of lipid metabolism through phosphatidylcholine synthesis. Furthermore, GNMT has been shown to be reduced during hepatic injury and multiple genetic analyses of human patients have found that GNMT gene expression is repressed in both NASH and hepatocellular carcinoma patients [9,10,11,12,13]. Despite this, GNMT protein downregulation and its impact on the disease process are still not fully understood.

In addition to transmethylation reactions, AdoMet is a required precursor for polyamine catabolism. AdoMet can donate its aminopropyl group to putrescine, the basic polyamine building block, to produce spermine and spermidine. Polyamines are polycations that play a role in multiple cellular processes, including cellular proliferation, differentiation, and death. The role of polyamine metabolism has been indicated in several disease states, including cancer and obesity [14,15]. Previously, we have demonstrated the role of polyamine flux and acetyl-CoA utilization in lipid accumulation in adipose tissue [14,16,17]. To date, the role of polyamine metabolism has not been studied in the pathophysiology of NASH.

This study shows the reduction of liver GNMT protein in subjects with NASH compared to healthy controls. To further assess the effects of GNMT downregulation in NASH, we used a diet-induced NASH animal model to show that GNMT downregulation leads to a significant increase in AdoMet levels. We also show that GNMT downregulation reduces transmethylation activity and increases flux into polyamine metabolism using targeted proteomics and metabolomics. Polyamine flux generates reactive oxygen species (ROS) that increase lipid peroxidation product 4-hydroxynonenal (4-HNE). This study provides key insights into metabolic changes in the liver contributing to a cytotoxic environment and NASH pathogenesis.

## 2. Results

### 2.1. GNMT Is Downregulated in Human NASH

**Figure 1:**
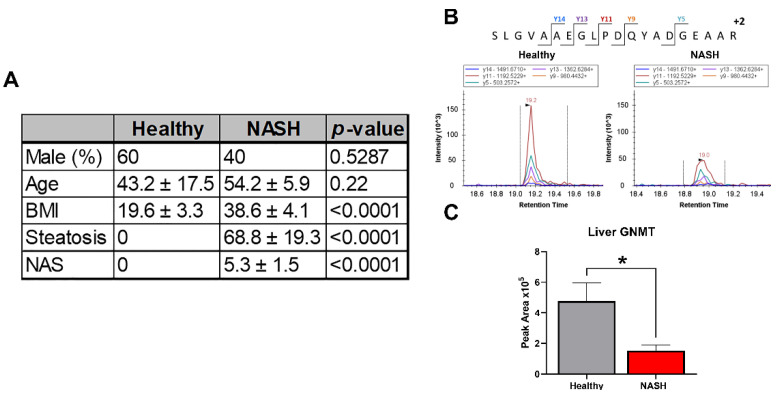
GNMT is downregulated in human NASH liver. (**A**) Table displaying patient characteristics for healthy and NASH liver samples. (**B**) Representative chromatograms from selected reaction monitoring analysis. (**C**) GNMT protein abundance in human liver samples. Data presented as mean ± SEM (N = 5, * *p* < 0.05).

Multiple studies have shown that GNMT mRNA levels are decreased in NASH subjects, and there is a negative correlation between the severity of liver damage and GNMT expression [10,11]. We developed and validated a targeted mass spectrometry method using selected reaction monitoring (SRM) to identify and quantitate liver GNMT in NASH and healthy subjects. The method had a coefficient of variation below 15% and 5% for technical and SRM replicates, respectively, and the peak area response was linear from 10 to 200 µg of total protein (R^2^ = 0.997) (Supplementary Figures S1 and S2). The control group consisted of 3 male and 2 female subjects with a mean age of 43 and a normal BMI. The NASH subjects included 2 males and 3 females with a mean age of 54. All NASH subjects had a BMI greater than 30 and were diagnosed with diabetes. We showed that GNMT liver protein levels were decreased threefold in NASH subjects compared to healthy subjects (Figure 1).

### 2.2. Modified Amylin Diet Induces NASH in Mice

**Figure 2:**
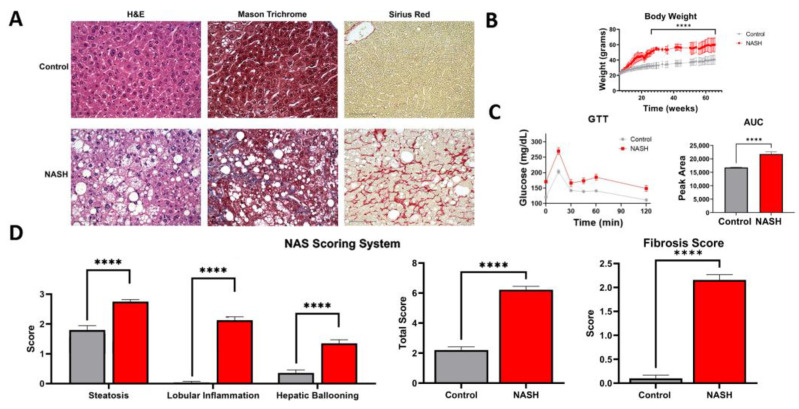
Modified amylin diet induces NASH in mice. (**A**) H&E, Masson trichrome, and Sirius red liver histology staining. Scale bar = 75 µm. (**B**) Body weight of mice. (**C**) Results from glucose tolerance test and area under the curve quantitation. (**D**) The NAS scoring system was used to assess liver histology. Mice fed a modified amylin diet were diagnosed with NASH with a total average NAS score of 6 and fibrosis score of 2. Data presented as mean ± SEM. (N = 7, **** *p* < 0.0001).

To better understand the effect of GNMT reduction in NASH progression, we modified a diet-induced animal model for studying NASH pathophysiology. Specifically, mice were fed a high-fat and fructose diet for 27 weeks to induce insulin resistance. Thereafter, cholesterol was added to the diet to mimic the consequences of metabolic syndrome in humans. Recent studies have shown that high-fat diets with high cholesterol can induce inflammation and fibrosis in rodent liver [18,19] and transcriptomic and metabolic analyses show that these diets can accurately model human NASH and activate key pathways involved in human NASH progression [9,20]. The modified amylin diet-fed mice showed a significant increase in weight compared to chow-fed mice and demonstrated insulin resistance as determined by glucose tolerance test (Figure 2B,C). The liver tissue was collected at the end of the study and liver tissue structure, integrity, and fibrosis were visualized by H&E, Mason trichrome, and Sirius red staining (Figure 2A). Tissue damage and cell death were evident in the modified amylin diet mice from fat deposition, cell enlargement, and immune cell infiltration. The NAFLD activity score (NAS) and fibrosis score were used to determine the extent of NASH pathophysiology [21,22]. The NAS scoring system is based on steatosis, lobular inflammation, and hepatic ballooning. The modified amylin diet mice had significantly higher levels of all three parameters compared to control and an average NAS score of 6. The degree of fibrosis was assessed by Mason trichrome and Sirius red staining of collagen fibers. The modified amylin diet mice had an average fibrosis score of 2 (Figure 2D). This analysis verified the diagnosis of NASH with moderate fibrosis in modified amylin diet mice.

### 2.3. Proteomic Characterization of NASH Animal Model Resembles Human Pathophysiology

**Figure 3:**
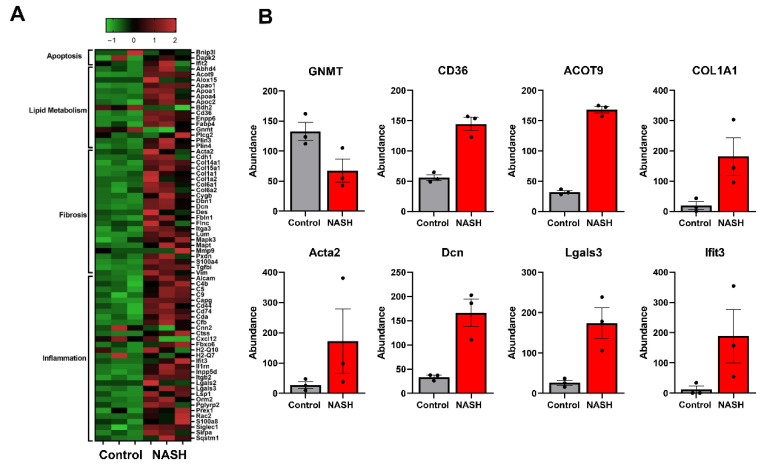
Proteomic characterization of NASH animal model shows human pathophysiology. (**A**) Heat map displaying differentially regulated proteins involved in apoptosis, lipid metabolism, fibrosis, and inflammation. (**B**) Individual bar graphs for markers of NASH. Glycine N-methyltransferase (GNMT), platelet glycoprotein 4 (CD36), acyl-coenzyme A thioesterase 9 (Acot9), collagen type 1 alpha 1 chain (COL1A1), actin alpha 2 (Acta2), decorin (Dcn), galectin-3 (Lgals3), interferon-induced protein with tetratricopeptide repeats 3 (Ifit3).

**Figure 4:**
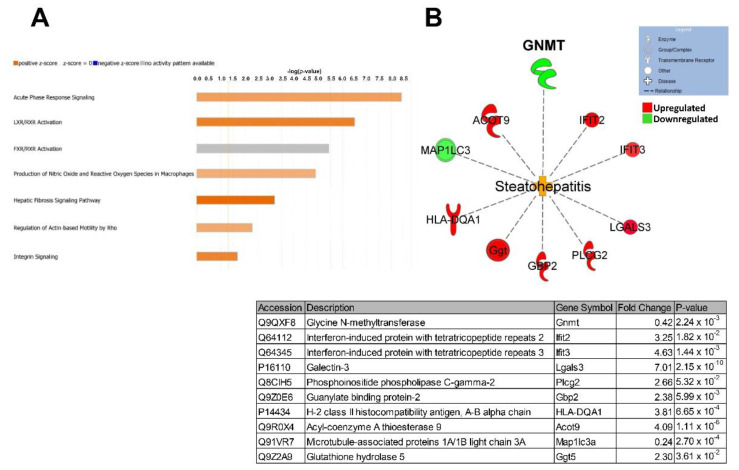
Ingenuity pathway analysis predicts activation of steatohepatitis. (**A**) Top altered canonical pathways in NASH liver. (**B**) Steatohepatitis network of differentially expressed proteins contributing to the disease state. Individual proteins are listed in the table below.

We then used global unbiased label-free proteomics to gain insight into NASH pathophysiology at the molecular level. Liver tissue from three control and three NASH mice was collected and individually processed for proteomic analysis. Our modified in-stage tip (iST) sample preparation process allows for minimal sample handling and fractionation to ensure high reproducibility and sensitivity [23,24,25,26]. From this analysis, a total of 4783 proteins were identified and quantified with high confidence (Supplementary Table S1). There were 646 differentially expressed proteins between control and NASH groups, 418 downregulated and 228 upregulated. Several differentially regulated proteins are involved in NASH pathophysiology, including lipid metabolism, apoptosis, fibrosis, and inflammation (Figure 3). Specifically, GNMT, platelet glycoprotein-4 (CD36), and acyl-coenzyme A thioesterase 9 (Acot9) are involved in lipid metabolism and have previously been associated with human NAFLD progression [7,27,28,29]. Collagen type 1 alpha 1 chain (COL1A1), actin alpha 2 (ACTA2), and decorin (Dcn) are three of the major markers of extracellular matrix deposition and liver fibrogenesis in humans [30]. Inflammatory and immunoregulatory proteins, galectin-3 (Lgals3) and interferon-induced protein with tetratricopeptide repeat 3 (Ifit3) were both upregulated in our NASH model in agreement with previous studies [31,32,33]. Currently galectin-3 inhibitors are being tested in the clinic to treat NASH [32]. We then used Ingenuity Pathway Analysis (IPA) software to analyze the differentially expressed proteins for overrepresented canonical pathways, regulators, and biological processes (Figure 4). IPA identified acute phase response signaling as the top altered canonical pathway, demonstrating immune response and inflammation in our animal model. Major nuclear receptor pathways were also identified as differentially regulated including liver X receptors (LXR), farnesoid X receptor (FXR), and retinoid X receptor (RXR). These nuclear receptors regulate liver metabolism and immunology and have been previously shown to induce steatohepatitis [34,35]. Several drugs targeting these nuclear receptors are under investigation for the treatment of NASH [36]. Furthermore, pathways involved in fibrosis signaling and oxidative stress production were shown to be activated in our animal model. Overall, these findings correlate well with human NASH pathophysiology and validate our animal model for studying NASH on the molecular level. IPA identified proteins contributing to steatohepatitis based on previous reports and differential protein expression. Several of these proteins including GNMT, ACOT9, and LGALS3 have been shown to rescue/induce NASH through genetic knockouts [7,29,31]. In accordance with our initial human liver analysis, the downregulation of GNMT was identified as a contributing factor to steatohepatitis. Despite this, the effects of GNMT attenuation under pathophysiological conditions are still not well understood.

### 2.4. Dysregulated AdoMet Metabolism in NASH

**Figure 5:**
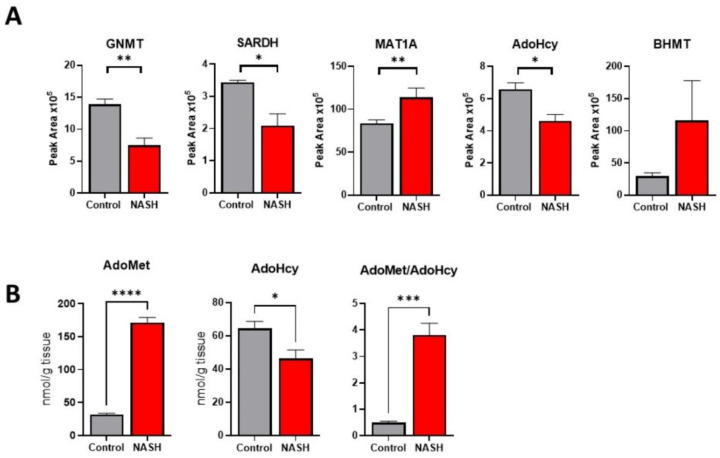
Dysregulation of AdoMet metabolism in NASH. (**A**) Relative protein abundance of select AdoMet regulating enzymes in control and NASH livers. (**B**) Levels of liver AdoMet and AdoHcy in control and NASH mice. Data presented as mean ± SEM (N = 4, **** *p* < 0.0001, *** *p* < 0.001, ** *p* < 0.01, * *p* < 0.05).

GNMT is highly abundant in hepatocytes and is the main regulator of AdoMet levels in the liver. Next, using a new group of control and NASH mice (N = 4), we wanted to confirm the downregulation of GNMT and further investigate the effect on AdoMet regulation. A targeted mass spectrometry method was developed and validated to quantify proteins involved in AdoMet regulation (Supplementary Table S2 and Supplementary Figures S1–S3). We found that both GNMT and sarcosine dehydrogenase (SARDH) were downregulated in NASH. Together, GNMT and SARDH form a futile cycle in the methylation and demethylation of glycine and sarcosine to control AdoMet concentration. Furthermore, we found that adenosylhomocysteinase (AHCY), an enzyme downstream of GNMT in the transmethylation pathway, was downregulated and that AdoMet biosynthetic enzyme, methionine adenosyltransferase 1A (MAT1A), was significantly upregulated in NASH (Figure 5A). We then used targeted metabolomic studies to quantitate the levels of AdoMet and S-adenosylhomocysteine (AdoHcy) in the liver. There was a fourfold increase in AdoMet levels and an approximately 30% reduction in AdoHcy in NASH (Figure 5B). This resulted in a significant increase in the AdoMet/AdoHcy ratio. These results show that AdoMet regulation is disrupted in NASH, inducing significantly higher concentrations of AdoMet.

### 2.5. Polyamine Metabolism Is Activated in NASH, Causing Flux and Oxidative Stress

**Figure 6:**
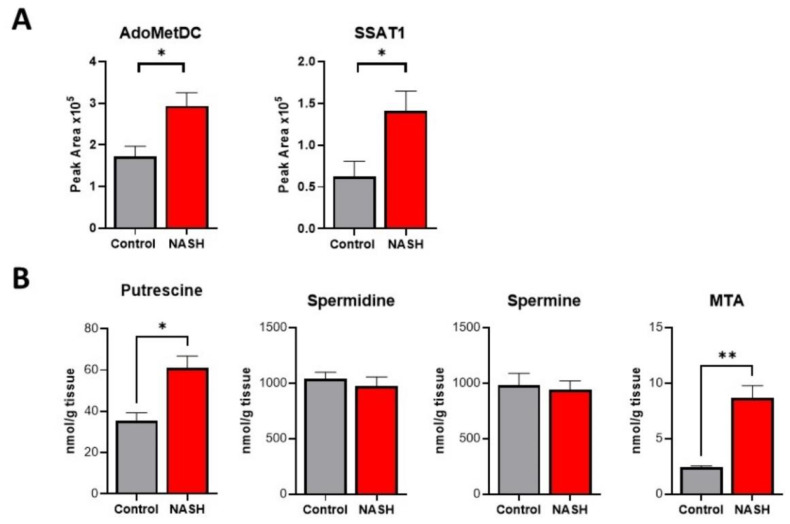
Polyamine metabolism is activated in NASH causing a flux. (**A**) Relative protein abundance of select polyamine metabolic enzymes in control and NASH livers. (**B**) Quantification of polyamine metabolites in control and NASH livers. Data presented as mean ± SEM (N = 4, ** *p* < 0.01, * *p* < 0.05).

**Figure 7:**
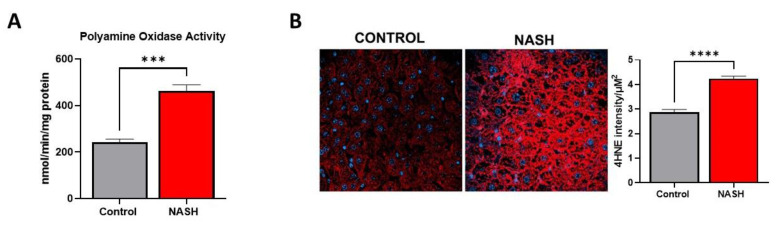
Increased activity of polyamine oxidase and oxidative damage in NASH. (**A**) Activity of polyamine oxidase in control and NASH livers. (**B**) Immunofluorescence staining of control and NASH livers for 4-hydroxynonenal modified proteins. Quantification of the fluorescence is shown on the right. Data presented as mean ± SEM (N = 4, **** *p* < 0.0001, *** *p* < 0.001).

In addition to transmethylation, AdoMet is a required substrate for polyamine synthesis, and its levels can impact polyamine flux. Based on the increased levels of AdoMet, we predicted discovering activation of polyamine metabolism. Targeted mass spectrometry analysis found a significant increase in both rate-limiting biosynthetic enzyme S-adenosylmethionine decarboxylase (AdoMetDC) and catabolic enzyme spermidine/spermine-N1-acetyltransferase (SSAT1) (Figure 6A). Quantification of key polyamine metabolites revealed a significant increase in the polyamine building block putrescine. Our analysis showed no change in spermidine and spermine levels, but a significant increase in 5′-methylthioadenosine (MTA), the by-product of spermidine and spermine synthesis (Figure 6B). Polyamine catabolism involves acetylation of spermine and spermidine by SSAT1 and acetyl CoA. The acetylated products can then be exported from the cell or oxidized to produce putrescine, hydrogen peroxide, and reactive aldehyde species. We found that the activity of catabolic enzyme polyamine oxidase (PAO) was significantly increased in NASH (Figure 7A). Together, these findings demonstrate an increased polyamine flux and rapid export or degradation back to putrescine. High rates of polyamine metabolism and polyamine oxidase activity can create high levels of oxidative stress by producing hydrogen peroxide and reactive aldehyde species. The degree of oxidative damage in the liver was measured using a monoclonal antibody for proteins modified by 4-hydroxynonenal (4HNE). 4HNE is a lipid peroxidation product and can modify biological molecules through carbonylation. We found significantly higher amounts of 4HNE carbonylation in NASH mice demonstrating a high degree of oxidative stress (Figure 7B). These findings are further supported by our global proteomics analysis showing that several proteins involved in redox potential are differentially regulated in NASH (Table S1). Among these proteins are glutathione peroxidase 3 and 5 and glutathione S-transferase theta 3, which have previously been indicated in NASH and are involved in 4HNE detoxification [37,38,39].

## 3. Discussion

Nonalcoholic steatohepatitis is a largely heterogeneous disease with a complex natural history. One of the major barriers to developing new therapies for NASH is the lack of clinically relevant in vitro or animal models. Over 70% of NASH patients are obese or have type II diabetes. Using a modified amylin diet-based murine model, we successfully replicated key features of human NASH associated with metabolic syndrome. The animals developed steatosis, lobular inflammation, and liver fibrosis while gaining significant weight and becoming insulin resistant. Our global proteomic analysis provided an in-depth molecular characterization of the disease state and identified key biomarkers and pathways associated with human NASH pathogenesis, including the downregulation of GNMT. We verified the reduction of GNMT in human NASH liver samples and Ingenuity Pathway Analysis identified GNMT downregulation as a key driver in steatohepatitis. We then used targeted mass spectrometry to understand how GNMT downregulation affected AdoMet regulation in our NASH model. Our findings correlated well with previous studies using GNMT KO animals showing elevated levels of AdoMet [7].

Interestingly, the depletion of AdoMet through knockout of synthetic enzyme methionine adenosyltransferase 1 A (MAT1A) or a methionine-choline deficient (MCD) diet has also been shown to lead to NASH [40,41,42]. Multiple studies have reported deficient AdoMet levels in association with NASH and evidence supporting the supplementation of AdoMet for treatment of NASH [43,44,45]. Therefore, the progression of NASH is viable in the presence of either elevated or reduced levels of AdoMet [46]. One explanation for these conflicting findings could be the models used for studying NASH pathogenesis and their translation to human disease. The studies supporting the notion of AdoMet supplementation for NASH treatment rely heavily on data based on the MCD diet. Methionine deficiency leads to decreased levels of AdoMet and results in hypomethylation of DNA and reduced VLDL assembly and export [41,47,48]. Our study chose to use a modified amylin diet to induce NASH based on the close association of NASH with metabolic syndrome [3]. The modified amylin diet co-presents with obesity and insulin resistance, whereas the MCD diet induces weight loss. In addition, a recent study comparing the MCD and a Western diet concluded that the human NASH metabolic profile was better represented by a diet high in fat, carbohydrates, and cholesterol [20]. Therefore, we hypothesize that our findings using the modified amylin diet more closely resemble the pathophysiology seen in NASH patients. This is further supported by our findings of reduced GNMT expression in NASH and type II diabetes patients. The heterogeneity of NASH could explain another possibility for these differences. The progression of NAFLD to NASH is a complex process that results from several genetic and environmental factors [4]. Genetic studies analyzing NASH have identified subgroups of NASH patients with distinct genetic signatures. A study performed by Alonso et al. identified an “M subgroup” of NASH patients with a similar genetic signature to MAT1A KO mice. However, this subgroup accounted for less than half of the study population [49]. This study illustrates NASH heterogeneity and demonstrates that the MCD diet does not properly model most NASH patients. In addition, a recent meta-analysis of seven human NAFLD genetic data sets identified GNMT downregulation in a genetic signature of NAFLD progression, while MAT1A was not acknowledged [10]. Our study provides evidence that the pathogenesis of NASH in association with metabolic syndrome causes an increase in AdoMet. These findings are further supported by previous studies showing that a high-fat diet can lead to increased AdoMet levels in association with NASH. In a study done by Maria Del Bas et al., they used a high-fat diet with selenium and vitamin E deficiency to induce NASH in hamsters [50]. They found that AdoMet is significantly upregulated in NASH associated with metabolic syndrome and not altered in simple steatosis. These findings can help stratify the highly heterogenous NASH population into subgroups based on their metabolic profile. The ability to personalize treatment for NASH will greatly help the success of phase three trials and the development of new therapies for NASH.

From our results, a consequence of elevated AdoMet levels was the activation of polyamine metabolism in NASH. AdoMet is a required substrate for polyamine synthesis, and high levels of AdoMet can cause a flux into polyamine metabolism. Both our proteomic and metabolomic analyses demonstrated the activation of polyamine metabolism in NASH, showing increased expression and activity of metabolic enzymes and both polyamine precursors and products of catabolism. These findings are supported by previous studies characterizing GNMT KO mice. Hughey et al. showed that the knockout of GNMT caused an increase in several polyamine metabolites to reduce elevated AdoMet levels [8]. From our understanding, this is the first time that polyamine flux has been shown in diet-induced NASH pathogenesis. Intracellular concentrations of polyamines can reach millimolar levels, and catabolism through polyamine oxidase can produce an overwhelming amount of hydrogen peroxide and lipid peroxidation products [51]. Polyamine flux has previously been indicated in several disease states including hepatocellular carcinoma and hepatitis C, causing oxidative stress and inflammation in the liver [52,53]. Furthermore, mechanistic studies performed in vivo and invitro have shown that inhibition of polyamine oxidation can reduce levels of reactive oxygen species and prevent cytotoxicity [52,54,55,56]. Oxidative stress, specifically lipid peroxidation, is strongly associated with NASH and is a driver of NAFLD progression [57,58]. Increased oxidative stress can lead to mitochondrial dysfunction, endoplasmic reticulum stress, and inflammation in NASH development [59]. This study shows that lipid peroxidation stress marker, 4HNE, was significantly increased in our NASH model. These findings highlight the contribution of polyamine metabolism to the production of oxidative stress. Further studies are required to assess the effect of inhibiting polyamine oxidation on NASH progression. Overall, this study shows that the reduction of GNMT in NASH pathophysiology results in an accumulation of AdoMet and polyamine flux leading to oxidative stress production.

## 4. Materials and Methods

### 4.1. Human Liver Samples

Human liver tissue was purchased commercially from XenoTech. Liver tissue was supplied as frozen prelysate in buffer.

### 4.2. Animal Studies

All animal experiments were approved by the Institutional Animal Care and Use Committee (IACUC). Specific pathogen-free, 6-week-old, male, C57BL/J6 mice of approximately 25 g body weight were purchased from Jackson’s laboratories, Bar Harbor, ME. Seven days after arrival, the animals were weighed and ear-tagged for individual identification. Animals were randomly assigned into two groups (N = 7 per group), with no statistical difference in body weight. At 7 weeks of age, the NASH group started receiving a high fat/high sucrose diet (Research Diets # D12331i) and at 28 weeks of age the diet was switched to a high fat/high fructose/high cholesterol diet (Research Diets # D09100310i). Control animals received a CHOW diet. Bodyweight was monitored weekly. A glucose tolerance test was performed at week 65. Animals were fasted for 5 h and administered a bolus of glucose (1 g/kg) by oral gavage. Blood samples were collected from the tip of the tail and blood glucose was measured at 0, 15, 30, 45, 60, and 120 min after the glucose administration. Animals were euthanized at 66 weeks of age and liver tissue was stored at −80 °C.

### 4.3. Histology and NAS Scoring

Formalin-fixed liver tissues were paraffin-embedded and sectioned onto slides. Slides from each animal were stained with hematoxylin and eosin (H&E), Mason trichrome, and Sirius red staining. All images were taken at 40× magnification. H&E and Sirius red staining were used to quantitate the NAFLD activity score (NAS) and fibrosis score, respectively. The scores were quantitated based on the scale developed by the Non-alcoholic Steatohepatitis Clinical Research Network. The parameters evaluated were steatosis, lobular inflammation hepatic ballooning, and fibrosis [60].

### 4.4. Global Mass Spectrometry Analysis

Proteins were extracted for label-free global proteomics studies by adding a 6 M guanidium hydrochloride buffer and dilution buffer (25 mM Tris, 10% acetonitrile). The proteins were digested with Lys-C for 4 h at 37 °C. Second digestion was achieved by overnight incubation with trypsin. The incubated solution was acidified and centrifuged at 4500× *g* for 5 min. The supernatants consisting of peptides were loaded onto activated in-house-made cation stage tips [25,26]. The peptides from each sample were eluted into six fractions using elution buffers as previously described [23,24,25,26]. Mass spectrometry analyses were performed on these fractions using the Q Exactive mass spectrometer (ThermoFisher Scientific, Waltham, MA, USA). The desalted tryptic peptide samples were loaded onto an Acclaim PepMap 100 pre-column (75 μm × 2 cm, ThermoFisher Scientific, Waltham, MA, USA) and separated by Easy-Spray PepMap RSLC C18 column with an emitter (2 μm particle size, 15 cm × 50 μm ID, ThermoFisher Scientific, Waltham, MA, USA) by an Easy nLC system with Easy Spray Source (ThermoFisher Scientific, Waltham, MA, USA). To elute the peptides, a mobile-phase gradient is run using an increasing acetonitrile concentration. Peptides were loaded in buffer A (0.1% (*v*/*v*) formic acid) and eluted with a nonlinear 145-min gradient as follows: 0–25% buffer B (15% (*v*/*v*) of 0.1% formic acid and 85% (*v*/*v*) of acetonitrile) for 80 min, 25–40% B for 20 min, 40–60% B for 20 min, and 60–100% B for 10 min. The column was then washed with 100% buffer B for 5 min, 50% buffer B for 5 min, and re-equilibrated with buffer A for 5 min. The flow rate was maintained at 300 nLmin.

Electron spray ionization was delivered at a spray voltage of 1500 V. MS/MS fragmentation was performed on the five most abundant ions in each spectrum using collision-induced dissociation with dynamic exclusion (excluded for 10.0 s after one spectrum), with automatic switching between MS and MS/MS modes. The complete system was entirely controlled by Xcalibur software 4.1. Mass spectra processing was performed with Proteome Discoverer v2.5. The generated de-isotoped peak list was submitted to an in-house Mascot server 2.2.07 for searching against the Swiss-Prot database (Release 2013_01, version 56.6, 538,849 sequences) and Sequest HT database. Both Mascot and Sequest search parameters were set as follows: species, homo sapiens; enzyme, trypsin with maximal two missed cleavage; fixed modification, cysteine carboxymethylation; 10 ppm mass tolerance for precursor peptide ions; 0.02 Da tolerance for MS/MS fragment ions.

### 4.5. Bioinformatic Analysis

Differentially expressed proteins from the proteomic analysis were further analyzed using Qiagen’s Ingenuity Pathway Analysis (IPA). Differentially expressed proteins were uploaded to IPA and compared against the Ingenuity Knowledge Base reference set. Overrepresented canonical pathways, diseases and functions, and biological networks were identified, and Fisher’s exact test was used to determine significance.

### 4.6. Polyamine Metabolomic Analysis

Polyamine metabolites were measured by HPLC using the AccQ.Fluor kit (Waters, Milford, MA, USA) described previously [61]. Polyamine oxidase activity was measured using soluble protein extracts of mouse liver tissue as previously described [17].

### 4.7. Targeted Mass Spectrometry Analysis

Liver tissue samples were prepared as described in global mass spectrometry analysis. Protein quantification was performed on a TSQ Quantum Ultra triple quadrupole mass spectrometer (Thermo Scientific, Waltham, MA, USA) equipped with an Ultimate 3000 RSLCnano system with autosampler (Thermo Scientific, Waltham, MA, USA). The mobile phase consisted of Buffer A, 0.1% formic acid and Buffer B, 85% acetonitrile with 0.1% formic acid. Peptides were separated on an Acclaim PepMap RSLC C18 precolumn (3 µm, 100 Å) and column (2 µm, 100 Å). They were then eluted with a flow gradient of 2–95% Buffer B over 20 min. Peptides entered the mass spectrometer through nanoelectrospray ionization with a voltage of 1600 V and capillary temperature 270 °C. The mass spectrometer was run in selected reaction monitoring (SRM) mode detecting the transitions listed in Table S2. Each protein was identified by at least two unique peptides. The selection of peptides for the SRM assay was based on the following: (1.) Peptides uniquely representing the target protein; (2.) no missed tryptic cleavage sites; (3.) peptide sequences with 6–25 amino acids; and (4.) amino acids susceptible to chemical modifications (Cys, Met) avoided. Peptide transitions were identified based on elution profile and fragmentation pattern compared to a spectral library (dotp) generated from our global proteomic analysis or the deep neural network, Prosit [62]. Each peptide was identified by the co-elution of at least five product ions. The reproducibility and linearity of each transition were also assessed in a set of replicate liver digests (Figures S1 and S2). The peptide with the smallest coefficient of variation and greatest coefficient of determination was used for quantification. All peptides used for quantification had a coefficient of variation <20% and demonstrated linearity from 10 to 200 µg of total protein. Data was collected using Xcalibur software 4.1 and imported into Skyline software 21.1 for peak area integration and data analysis.

### 4.8. HNE Immunofluorescence

Liver sections were subjected to immunofluorescence to evaluate 4HNE-modified protein expression. Briefly, slides were deparaffinized and epitopes were retrieved with citric acid solution followed by incubation in glycine solution. The non-specific background was blocked using 10% goat serum. Slides were incubated with anti-4HNE monoclonal antibody (ab46545 Abcam, Cambridge, England), washed, and incubated again with goat anti-rabbit antibody (Thermo Fisher A27034). Nuclei were stained with DAPI. Pictures were taken using confocal microscopy and 4HNE intensity was quantitated with Harmony software 4.8.

### 4.9. Statistical Analysis

Data are expressed as mean values ± standard error of the mean (SEM). Statistical comparisons were performed using unpaired, two-tailed Student’s *t*-tests. Differences were considered significant when *p* < 0.05.

## Data Availability

The mass spectrometry proteomics data have been deposited to the ProteomeXchange Consortium via the PRIDE partner repository with the dataset identifier PXD030781 and 10.6019/PXD030781.

## Supplementary Materials

The following supporting information can be downloaded at: https://www.mdpi.com/article/10.3390/ijms23041986/s1.
