# Supplementary material for: Dysregulation of S-adenosylmethionine Metabolism in Nonalcoholic Steatohepatitis Leads to Polyamine Flux and Oxidative Stress
Source: Int J Mol Sci. 2022 Feb 11;23(4):1986. doi: 10.3390/ijms23041986 (PMC8878801; doi:10.3390/ijms23041986)
Supplement: Supplementary file 1 [file ijms-23-01986-s001.zip › NASH polyamine_SuppFig.pdf]

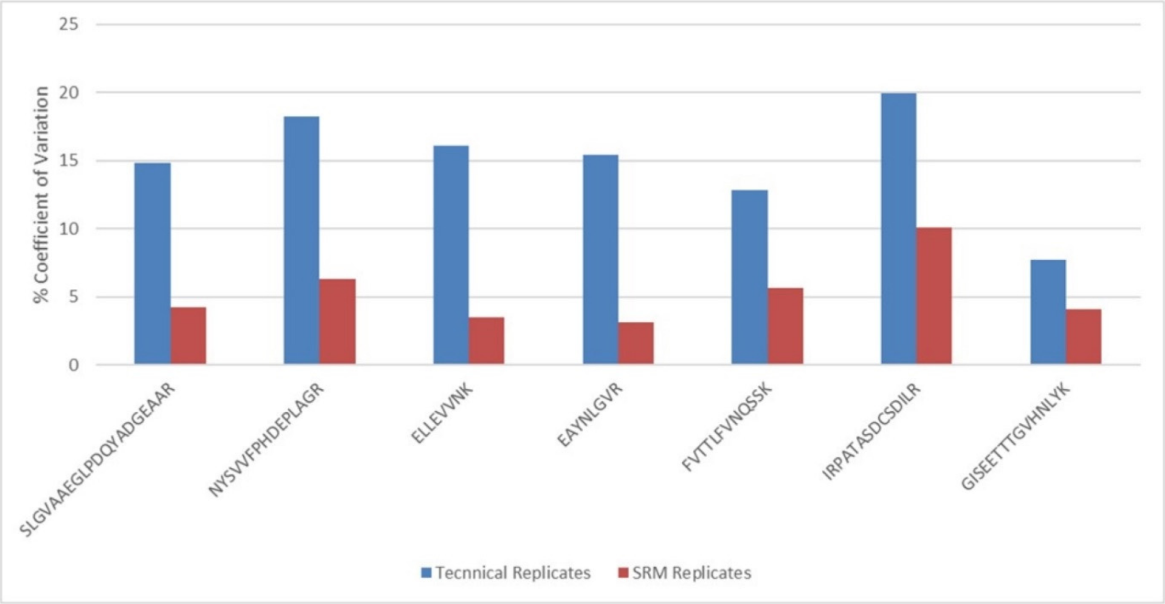

Figure S1. SRM peptide coefficient of variation. Five replicate preparations of liver protein lysate were prepared to measure the technical replicate coefficient of variation for each peptide. The SRM replicate variation was assessed by three replicate injections of the same sample preparation. All peptides used for quantification had a coefficient of variation below 20%.

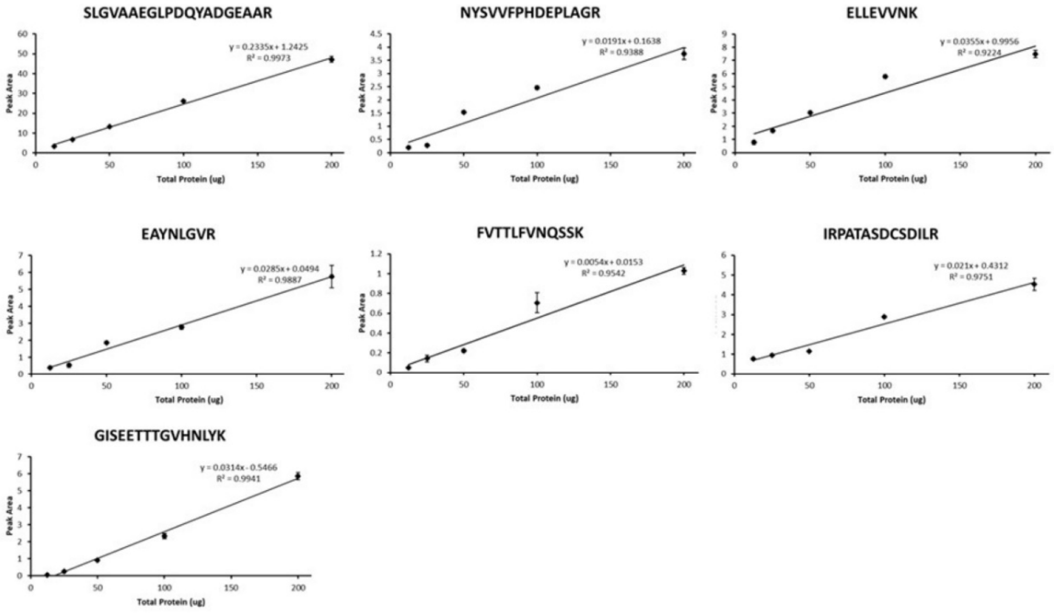

Figure S2. SRM peptide linearity. The linearity of each peptide assay was assessed by preparing liver protein lysate samples with varying amounts of total protein amount and measuring peptide transitions. Samples were prepared in triplicate. The linear regression for each peptide is shown and a total of 100ug of protein was used for subsequent analyses. Data presented as mean ± SEM.

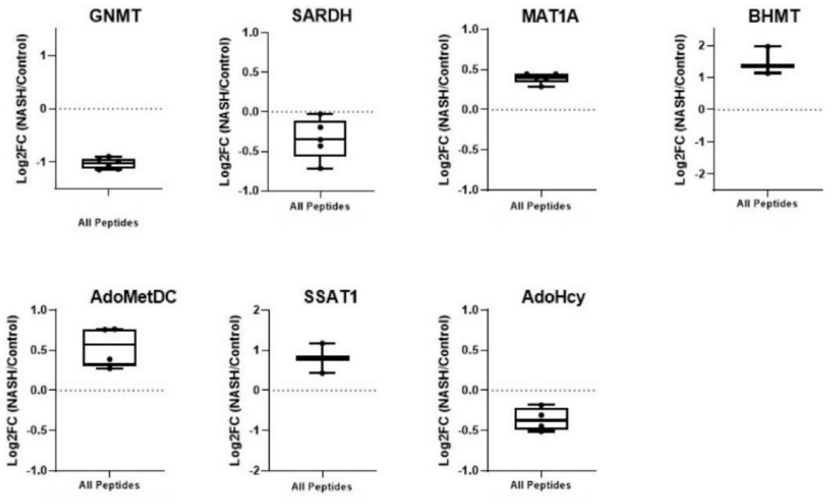

Figure S3. SRM quantitation of all peptides. Box plots showing the log2 fold change between control and NASH liver for all peptides used for the identification of each protein. The peptide with the lowest coefficient of variation and highest coefficient of determination was used for protein quantification.
